# Parenting in the early years and self-harm in adolescence: The role of control and reward systems in childhood

**DOI:** 10.1016/j.jad.2023.07.061

**Published:** 2023-10-15

**Authors:** E. Dawe-Lane, E. Flouri

**Affiliations:** a Institute of Cognitive Neuroscience, University College London, London, United Kingdom; b Institute of Education, Psychology and Human Development, University College London, London, United Kingdom

**Keywords:** Self-harm, Parenting, Emotion regulation, Decision-making, Reward processing

## Abstract

**Background:**

Research suggests that early parenting may contribute to the development of self-harm but this has not been examined longitudinally. In this study, we explored the relationship between early parenting and self-harm in adolescence and considered whether (1) emotion regulation and (2) decision-making in childhood mediate the relationship between early parenting and self-harm.

**Method:**

Using longitudinal data from the Millennium Cohort Study (MCS), we tested mediation models exploring the relationship between early parenting and self-harm in adolescence via emotion regulation and decision-making. Parenting was assessed at age 3 with measures of conflict, closeness and discipline. The trajectories of independence & self-regulation and emotional dysregulation were modelled from ages 3 to 7 years through latent growth curve analysis, with individual predicted slope and intercept values used in the mediation models. Decision-making (deliberation time, total time, delay aversion, quality of decision making, risk adjustment, risk-taking) was assessed using the Cambridge Gambling Task (CGT) at age 11.

**Results:**

In our sample (*n* = 11,145), we found no evidence of a direct association between early parenting and self-harm in adolescence. However, there were indirect effects of parenting (conflict and closeness) on self-harm via the slope of emotional dysregulation. Furthermore, delay aversion was positively associated with self-harm in adolescence.

**Limitations:**

It must be acknowledged that we cannot determine causality and that self-report measures of parenting are vulnerable to several biases.

**Conclusion:**

The findings support early identification and interventions for children exhibiting chronic emotional dysregulation and decision-making characterised by a bias for smaller, immediate over larger, delayed rewards.

## Highlights

• Indirect effects of early parenting on adolescent self-harm via chronic emotional dysregulation in childhood.
• Delay aversion in childhood is associated with self-harm in adolescence.
• Findings support interventions for chronic emotional dysregulation and delay aversion in childhood.

## 1 Introduction

### 1.1 Self-harm

Self-harm can be defined as any behaviour undertaken to inflict damage to oneself, including cutting, scratching, burning, banging/hitting and poisoning, irrespective of suicidal intention (Hawton et al., 2003; Sornberger et al., 2012). Adolescence is a critical period, with the average age of onset of self-harm between 12 and 16 years (Plener et al., 2015). Research has shown that approximately 1 in 6 adolescents have self-harmed in their lifetime (Muehlenkamp et al., 2012), with reported lifetime prevalence between 10 and 25 % in the community (Baetens et al., 2011; Mahl et al., 2014; Muehlenkamp et al., 2012; Swannell et al., 2014), and 19 and 82.4 % in clinical samples (Nock and Prinstein, 2004; Selby et al., 2012). There is empirical evidence to suggest that rates of self-harm are increasing among adolescents and young adults (Klonsky et al., 2014), which is concerning as self-harm is a strong predictor of future suicide attempts (Hawton et al., 2012; Whitlock et al., 2013; Wilkinson et al., 2011) and has been associated with a range of mental health conditions including depression, anxiety, attention-deficit/hyperactivity disorder (ADHD), borderline personality disorder (BPD), and suicide (Crowell et al., 2012; Willoughby et al., 2015). As such, the early identification and prevention of self-harm is a research priority, however, existing interventions are typically initiated in adolescence, and after individuals have engaged in self-harm behaviours (Beauchaine et al., 2019). If self-harm is to be prevented, it will be critical to gain a better understanding of the risk factors for self-harm to support the early identification of vulnerable individuals and to develop efficacious prevention and intervention strategies.

### 1.2 Parenting and emotion regulation

Self-harm is considered the result of a complex interplay between genetic, biological, psychological, social, and cultural factors (Hawton et al., 2012). Some research has postulated that parenting has a role in the development and maintenance of self-harm through mediating factors such as poor emotion regulation (Adrian et al., 2011; Buckholdt et al., 2009) and maladaptive coping strategies (Glazebrook et al., 2016). One prominent theory (Linehan, 1993) posits that self-harm arises as a result of i) heightened sensitivity to emotional stimuli, ii) difficulty regulating intense emotions, and iii) slow return to emotional baseline. It is thought that invalidating relationships with parents, where the individual's emotions are neglected or ignored, contribute to emotional dysregulation, which in turn increases the likelihood of engaging in self-harm to cope with emotional distress (Crowell et al., 2009; Linehan, 1993; Wolff et al., 2019). Parents play a fundamental role in modelling how to moderate and manage emotion, through appropriate behavioural and emotional responses, until a child's emotional regulation is internalised (Eisenberg et al., 1998). Therefore, parenting environments that fail to provide adequate socialisation of emotion and support are likely to contribute to emotional dysregulation (e.g., limited ability to modulate and manage emotions), increasing the risk of self-harm in adolescence as a maladaptive strategy to cope with negative emotions such as sadness, anger, and guilt (Klonsky, 2007).

Parallel lines of research suggest that harsh and critical parenting is associated with poor emotion regulation (Morris et al., 2017; Morris et al., 2007), externalising problems (McKee et al., 2008), internalising problems (Gorostiaga et al., 2019) and self-harm (see (Fong et al., 2021) for a review) in adolescence. For example, (Adrian et al., 2011) found direct influences of perceived parenting (conflict and lack of support) on self-harm in adolescence, and that the relationship between parenting and self-harm was mediated by emotional dysregulation. That said, while there is a plethora of evidence from cross-sectional studies that parenting environments that are low on support and care (Ammerman and Brown, 2018; Baetens et al., 2014, Baetens et al., 2015; Boričević Maršanić et al., 2014; Brausch and Gutierrez, 2010; Claes et al., 2015; Emery et al., 2017; Martin et al., 2011; Martin et al., 2016; Tschan et al., 2015), or high on control (Ammerman and Brown, 2018; Baetens et al., 2014; Boričević Maršanić et al., 2014; Martin et al., 2011; Martin et al., 2016; McLafferty et al., 2019; You et al., 2017) and conflict (Keenan et al., 2014; Victor et al., 2019) are associated with adolescent self-harm, emerging longitudinal data reveal that the relationship between parenting and self-harm is reciprocal, whereby knowledge of self-harm can modify parenting behaviour in adolescence (e.g., eliciting more parental control) (Baetens et al., 2015; You et al., 2017). Contemporaneously, theoretical models contend that early parenting contributes to the development and maintenance of self-harm (Chapman et al., 2006; Linehan, 1993), but longitudinal studies are needed to establish the temporal association between parenting and self-harm and to determine whether early parenting contributes to risk of self-harm in adolescence.

### 1.3 Decision-making and reward processing

Although self-harm is often examined in relation to emotion regulation, there has been an increased focus on cognitive phenomena, such as reward processing and decision-making, to understand self-harm. For example, impulsivity has frequently been linked with self-harm (Simeon et al., 1992) as it is typically risky, damaging and undertaken without prior planning (Lockwood et al., 2017). Nevertheless, previous research into impulsivity and self-harm is conflicting as people who self-harm regularly self-report impulsivity (Janis and Nock, 2009), but there is often no observable difference compared to controls in behavioural tasks (Hamza et al., 2015; Janis and Nock, 2009; McCloskey et al., 2012; Oldershaw et al., 2009), which may reflect differences in the definition and assessment of impulsivity across studies. That said, it should be noted that few behavioural studies have incorporated affective components, which is perhaps surprising as self-harm typically occurs in the context of negative emotion. Indeed, some recent research that has incorporated affective components has linked self-harm with impulsivity; demonstrating that people who self-harm show impulsive decision-making following the induction of negative mood (Allen et al., 2019). However, it is currently unclear whether decision-making differences are evident prior to the onset of self-harm.

Additionally, (Nock and Prinstein, 2004) propose that self-harm is positively and negatively reinforced by intrapersonal (e.g., to reduce negative affect or numbing) and interpersonal (e.g., to gain attention from parents and peers or avoid punishment) mechanisms (Nock, 2009), which indicates that reward processing is fundamental to the development and maintenance of self-harm. In accordance, researchers have argued that decision-making is biased towards the short-term rewards of self-harm behaviours (e.g., reduced negative affect or gaining attention), with people who self-harm typically discounting the long-term negative impacts (e.g., scarring) (Lutz et al., 2022). Self-harm is often viewed as the outcome of intrapersonal negative reinforcement, whereby it functions to allow individuals to avoid negative emotion and is reinforced by the reduction or avoidance of distress (Rasmussen et al., 2016). However, (Hilt et al., 2008) found support for a link with interpersonal positive reinforcement, with individuals engaging in self-harm in order to receive more support from others (e.g., parents and peers), yet research into the direct link between reward processing/decision-making and self-harm in adolescence is limited. Furthermore, while this theory can explain the relationship between parenting and self-harm in adolescence, where self-harm may be a way of avoiding punishment or communicating distress, engaged to elicit a response from others (e.g., parents) (Nock, 2008), it does not account for the role of early parenting. It could be that an early parenting environment that provides limited support and opportunities for young children to learn to express and manage their emotions results in aberrant reward processing and decision-making that in turn increases the risk of self-harm, but this has yet to be examined. Therefore, it will be important to investigate the relationship between factors such as early parenting, emotion regulation, decision-making and reward processing in our endeavour to understand the psychopathogenesis of self-harm in adolescence.

### 1.4 Aims

Research suggests that parenting, emotional dysregulation, and reward-processing and decision-making are associated with self-harm in adolescence, but the relationships have not been explored in a longitudinal design. Therefore, the present study aims to (i) explore the relationship between early parenting and self-harm in adolescence, (ii) determine whether emotion regulation, reward processing and decision-making in childhood are associated with self-harm in adolescence, and (iii) consider whether (1) emotion regulation and (2) reward processing and decision-making in childhood mediate the relationship between early parenting and self-harm in adolescence.

## 2 Methods

### 2.1 Millennium cohort study

Secondary data analysis was conducted on data from the Millennium Cohort Study (MCS); a large, longitudinal study exploring the development of children (*n* = 19,519) born in the United Kingdom at the start of the millennium. The first wave (MCS1) of data was collected from 18,552 families between 2001 and 2002, with a total of 18,818 infants aged between 9 and 11 months. The same sample was then invited to follow up (MCS2) when the children were 3 years old. 14,898 families from MCS1 were followed up and 692 new families were recruited into the cohort, totalling 15,590 families in the second wave (MCS2). 15,246 families were followed up in the third wave (MCS3), 13,857 in the fourth wave (MCS4), 13,287 in the fifth wave (MCS5), 11,714 in the sixth wave (MCS6), and 10,625 in the seventh wave (MCS7), when cohort members were 5, 7, 11, 14 and 17 years old, respectively.

The present study utilises data from 6 sweeps (MCS1-MCS6), including data from when cohort members were around 9 months, 3, 5, 7, 11, and 14 years old. Our final sample included 11,145 families with cohort members that completed the self-harm measure included in the self-report questionnaire administered at age 14. For the few families with twins or triplets in the study, we included only the first-born twin or triplet, thus avoiding the need for additional levels of analysis accounting for intra-family variability. In addition, we only included information provided by the main parent, excluding partner and proxy partner datasets.

### 2.2 Measures

#### 2.2.1 Parenting

##### 2.2.1.1 Conflict and closeness

Parents were asked about their relationship with their child and about their child's behaviour using the Child-Parent Relationship Scale (CPRS Short Form) (Pianta and Steinberg, 1992; Pianta et al., 1995) at age 3. The scale consists of 15 items assessing two dimensions, conflict (8 items; reverse-scored) and closeness (7 items), which can be summed to produce total scores reflecting the extent to which there is a positive relationship between parent and child. Each item had 5 possible responses that were numerically coded: (1) definitely does not apply, (2) not really, (3) neutral, not sure, (4) applies sometimes, and (5) definitely applies.

##### 2.2.1.2 Discipline

In addition, parents were asked about their discipline practices at age 3. The measure consisted of 7 items in MCS adapted from the Conflict Tactics Scale (Straus et al., 1998), which was developed to measure the strategies that parents utilise to discipline their children. The items included in the MCS were ‘how often do you ignore/smack/shout/send to their bedroom or naughty chair/take away treats/tell off/bribe with sweets or other… when cohort member is naughty?’. Each item had 6 possible responses that were numerically coded: (0) never, (1) rarely, (2) once a month, (3) at least once a week, (4) daily, and (5) can't say. This was re-coded to create a scale ranging from (0) never to (4) daily. The Cronbach's alpha value (0.70) supported the 7 items being summed to create a discipline score (ranging from 0 to 21).

#### 2.2.2 Emotion regulation

The Child Social Behaviour Questionnaire (CSBQ) (Melhuish et al., 2004), completed by parents at ages 3, 5 and 7, was used as an indicator of emotion regulation. The questionnaire consists of 15 items and three sub-scales: (i) independence and self-regulation, (ii) emotional dysregulation, and (iii) cooperation (only included at age 7). Parents were asked whether the cohort member: ‘likes to work things out for self, does not need much help with tasks, chooses activities on their own, persists in the face of difficult tasks, moves to a new activity after finishing a task’ (i), ‘shows mood swings, gets over excited, is easily frustrated, gets over being upset quickly (reverse coded), acts impulsively’ (ii), and ‘is calm and easy going, works/plays easily with others, says please and thank you when reminded, waits his/her turn in games/activities, co-operates with requests’ (iii). Each item has 4 possible responses that were numerically coded: (1) not true, (2) somewhat true, (3) certainly true, and (4) can't say. We used independence and self-regulation and emotional dysregulation scores at ages 3, 5 and 7 to calculate trajectories of emotion regulation in childhood.

#### 2.2.3 Decision-making and reward processing

The Cambridge Gambling Task (CGT), administered for the first time in the MCS at age 11 years, was used to measure reward processing and decision-making. In a series of five stages administered during interview, the cohort member was presented with a row of 10 red or blue boxes across the top of the screen, which appeared in varying combinations. During the first (decision-only) stage the participant was asked to guess whether a yellow token was hidden in a red or a blue box. In the remaining four (gambling) stages, the participant selected a portion of 100 points allocated to them at the beginning of the trial to gamble on their confidence in the location of the token (and bets were presented in ascending and descending order). The ratios of red:blue boxes were presented in a pseudorandom order varying from 1:9 to 9:1. Therefore, the odds of guessing correctly were presented explicitly by varying the ratios of colours among boxes that may have contained the hidden token. Participants were informed that correct bets would be added to their points score and incorrect bets would be deducted, and that the objective was to win as many points as possible. Participants were asked to bet a proportion of their points (ranging from 5 % to 95 %) on the certainty of each decision. The first two stages were used for practice. As such, the cohort members' performance was assessed in the last two stages.

The CGT produces seven outcome measures: test duration (time taken for the individual to complete the task [seconds]), quality of decision-making (mean proportion of trials where the most likely outcome was selected), overall proportional bet (the mean proportion of points bet across all trials), deliberation time (mean time taken to make a box colour response [milliseconds]), delay aversion (difference in percentage bet in ascending versus descending conditions), risk adjustment (the extent to which betting behaviour was moderated by box ratio), and risk-taking (mean proportion of points bet on trials where the most likely outcome was chosen). Multicollinearity among CGT measures was assessed by inspection of the variance inflation factor (VIF) values. The risk-taking and the overall proportion bet variables were very highly intercorrelated (*r* > 0.90, *p* < .001), with a VIF of 1.00, and we therefore excluded overall proportion bet from further analyses.

#### 2.2.4 Self-harm

Self-harm was assessed at age 14 using a single item: “In the past year have you hurt yourself on purpose?” (yes, no).

#### 2.2.5 Confounders

The following variables were considered confounders (associated with both exposure and outcome) and measured at 9 months: sex (female, male), ethnicity (White, Non-white), birthweight (kgs), poverty (OECD 60 % median income indicator; above, below), siblings (number of siblings in the household), and family structure (two-parent, one-parent). In addition, we controlled for parent mental health (Malaise Inventory; Rutter 1970), parent smoking status (smoker, non-smoker), and parent education (higher education, NVQ4+: yes, no). Furthermore, we controlled for whether or not the cohort member was showing evidence of puberty (yes, no) reported by parents at age 11 and self-reported depression (short moods and feelings questionnaire; (Angold et al., 1995)) at age 14. See supplementary file for further information on the confounding variables.

### 2.3 Statistical analysis

All analyses were performed in STATA (Stata Corporation, College Station, TX, 1997). We used latent growth curve modelling (Jung and Wickrama, 2008) to identify trajectories of (i) independence & self-regulation and (ii) emotional dysregulation from 3 to 7 years. The individual predicted values of the intercept (set at baseline) and slope (rate of change) were used in the models. We used the predict command to generate predictions for the out-of-sample cases (e.g., the cases that were not included in the original estimation). This command employs the maximum likelihood with missing values (MLMV) estimate for those who had data on at least one time-point and single imputation for those with missing data on all time-points. This allowed us to estimate the intercepts and slope values for the whole analytic sample (*n* = 11,145).

The outcome variable (self-harm) and emotion regulation mediator (slope and intercept of independence & self-regulation and emotional dysregulation) had complete data. Missingness in the remaining variables ranged from 3.51 (sex) to 26.7 % (discipline). Missing data were imputed (20 imputed datasets) using multiple imputation by chained equations (MICE) (White et al., 2011), with the assumption that missingness was dependent on observed data (missing at random). We used all model variables to predict the missing values. During imputation and analysis, the MCS sampling stratum was controlled to account for the MCS study design.

We fitted two sets of structural equation models (see supplementary file, Fig. 1, for a graphical representation) in the complete cases and imputed cases samples. Model 1 included the slopes and intercepts for independence & self-regulation and emotional dysregulation, and the variables for early parenting (conflict, closeness, and discipline) and CGT (test duration, delay aversion, deliberation time, quality of decision making, risk adjustment and risk-taking), predicting self-harm. Model 2 adjusted for confounders measured at 9-months (sex, ethnicity, birthweight, family structure, siblings, parent education, parent mental health and parent smoking status) and pubertal status measured at age 11.

## 3 Results

### 3.1 Descriptive statistics

A total of 1634 (14.7 %) individuals reported that they had self-harmed in the past 12-months at age 14. Individuals who self-harmed were more likely to be female (X^2^_(1)_ = 373.724, *p* < .001), White (X^2^_(1)_ = 37.765, p < .001), and from one-parent families (X^2^_(1)_ = 6.786, *p* = .009) at 9-months in addition, individuals who self-harmed at age 14 were more likely to have parents that had not completed higher education (X^2^_(1)_ = 5.768, *p* = .016) and were smokers (X^2^_(1)_ = 52.775, p < .001) at 9-months (supplementary file, Table 1). Table 1 shows the descriptive statistics including the means or percentages for all variables included in the models. See supplementary file, Table 2, Table 3, for a sample bias analysis.

**Table 1:** Descriptive statistics in the analytic sample (unweighted data).

	N	%
*Age 9-months*		
Sex		
Female	5431	50.5
Male	5323	49.5
Ethnicity		
White	8877	82.7
Non-White	1855	17.3
Household factors		
Poverty (OECD 60 % median income indicator)		
Above	7335	68.4
Below	3390	31.6
Family structure		
Two-parent	9289	86.4
One-parent	1466	13.6
Siblings		
0	4466	41.5
1	3789	35.2
2	1643	15.3
3+	857	8
Parent factors		
Education (higher education, NVQ 4+)		
Yes	3702	35.5
No	6725	64.5
Smoking Status		
Yes	2824	26.3
No	7921	73.7
*Age 14*		
Self-harm		
Yes	1634	14.7
No	9511	85.3


	N	M (SE)
*Age 9-months*		
Parent mental health (malaise)	10,339	1.76 (0.021)
*Age 3*		
Closeness	8684	33.63 (0.023)
Conflict	8811	17.05 (0.062)
Discipline	8173	19.61 (5.18)
Independence & self-regulation	9808	2.47 (0.003)
Emotional dysregulation	9810	1.87 (0.005)
*Age 5*		
Independence & self-regulation	10,230	2.53 (0.003)
Emotional dysregulation	10,230	1.70 (0.005)
*Age 7*		
Independence & self-regulation	10,033	2.52 (0.004)
Emotional dysregulation	10,035	1.70 (0.005)
*Age 11*		
Test duration	10,102	9.48 (0.047)
Deliberation time	10,095	3384.22 (30.153)
Delay aversion	10,057	0.306 (0.005)
Quality of decision making	10,095	0.779 (0.004)
Risk adjustment	10,094	0.513 (0.021)
Risk-taking	10,094	0.541 (0.004)

**Table 2:** Regression coefficients for self-harm in complete and imputes cases.

	Complete cases			Imputed cases		
	B	SE	95 % CI	B	SE	95 % CI
	Model 1 (*n* = 7036)			Model 1 (n = 11,145)		
*Emotion regulation*						
Slope of independence & self-regulation	−0.151	0.122	−0.391, 0.088	−0.016	0.090	−0.193, 0.162
Intercept of independence & self-regulation	0.073	0.039	−0.003, 0.149	**−0.068**⁎⁎	**0.031**	**0.008,** **0.128**
Slope of emotional dysregulation	**0.448**⁎⁎⁎	**0.112**	**0.229,** **0.667**	**0.391**⁎⁎⁎	**0.085**	**0.224,** **0.558**
Intercept of emotional dysregulation	0.039	0.026	−0.013, 0.090	0.034	0.020	−0.005, 0.074
*CGT*						
Risk-taking	**−0.179**⁎⁎⁎	**0.037**	**−0.251, −0.106**	**−0.177**⁎⁎⁎	**0.030**	**−0.236,** **−0.118**
Risk adjustment	−0.005	0.006	−0.017, 0.007	−0.002	0.005	−0.012, 0.008
Total time	−0.000	0.000	−0.000, 0.000	0.000	0.000	−0.000, 0.000
Delay aversion	0.045	0.024	−0.001, 0.092	**0.052**⁎⁎	**0.020**	**0.013,** **0.091**
Deliberation time	0.000	0.000	−0.000, 0.000	0.000	0.000	−0.000, 0.000
Quality of decision making	0.055	0.047	−0.039, 0.148	0.067	0.034	−0.000, 0.136
*Parenting*						
Conflict	0.001	0.001	−0.000, 0.003	**0.002**⁎	**0.001**	**0.000,** **0.004**
Closeness	0.003	0.002	−0.002, 0.009	0.003	0.002	−0.002, 0.007
Discipline	0.002	0.001	−0.001, 0.003	0.000	0.001	−0.001, 0.002


	Complete cases			Imputed cases		
	B	SE	95 % CI	B	SE	95 % CI
	Model 2 (*n* = 6525)			Model 2 (n = 11,145)		
*Emotion regulation*						
Slope of independence & self-regulation	−0.173	0.120	−0.409, 0.063	−0.074	0.088	−0.248, 0.099
Intercept of independence & self-regulation	0.027	0.039	−0.050, 0.104	0.016	0.029	−0.042, 0.074
Slope of emotional dysregulation	**0.472**⁎⁎⁎	**0.107**	**0.261,** **0.683**	**0.397**⁎⁎⁎	**0.079**	**0.243,** **0.552**
Intercept of emotional dysregulation	0.021	0.027	−0.031, 0.074	0.015	0.021	−0.026, 0.056
*CGT*						
Risk-taking	−0.065	0.037	−0.136, 0.008	−0.055	0.030	−0.115, 0.004
Risk adjustment	0.005	0.006	−0.007, 0.017	0.005	0.005	−0.005, 0.015
Total time	−0.000	0.000	−0.000, −0.000	−0.000	0.000	−0.000, 0.000
Delay aversion	**0.056**⁎⁎	**0.025**	**0.006,** **0.105**	**0.062**⁎⁎⁎	**0.019**	**0.024,** **0.101**
Deliberation time	0.000	0.000	−0.000, 0.000	0.000	0.000	−0.000, 0.000
Quality of decision making	0.040	0.048	−0.054, 0.135	0.044	0.034	−0.023, 0.111
*Parenting*						
Conflict	−0.000	0.000	−0.002, 0.002	0.001	0.001	−0.001, 0.003
Closeness	0.004	0.003	−0.137, 0.008	0.002	0.002	−0.002, 0.006
Discipline	**0.004**⁎⁎⁎	**0.001**	**0.002,** **0.006**	0.002	0.001	−0.001, 0.004

*Note*. Model 1 = Parenting, slopes and intercepts of independence & self-regulation and emotional dysregulation, and CGT.

Model 2 = Model 1 + sex, ethnicity, birthweight, family structure, siblings, poverty, parent education, parent mental health, parent smoking status, and puberty.

⁎ *p* < .05.

⁎⁎ *p* < .01.

⁎⁎⁎ *p* < .001.

**Table 3:** Mediation (by CGT) of parenting on self-harm in imputed cases.

Direct and indirect paths	Imputed cases (n = 11,145)		
	B	SE	95 % CI
**Risk-Taking**			
Discipline ➔ risk-taking	**0.0019**⁎⁎⁎	**0.0005**	**0.0009, 0.0029**
Conflict ➔ risk-taking	−0.0007	0.0005	−0.0018, 0.004
Closeness ➔ risk-taking	−0.0008	0.0012	−0.0032, 0.0016
Risk taking ➔ self-harm	−0.0555	0.0303	−0.1152, 0.0042
Discipline ➔ self-harm	0.0015	0.0012	−0.0009, 0.0038
Conflict ➔ self-harm	0.0008	0.0010	−0.0013, 0.0028
Closeness ➔ self-harm	0.0022	0.0021	−0.0019, 0.0064
Indirect effect with discipline as a predictor	−0.0001	0.0001	−0.0002, 0.0000
Total effect with discipline as a predictor	0.0014	0.0012	−0.0009, 0.0037
Indirect effect with conflict as a predictor	0.0001	0.0001	−0.0000, 0.0001
Total effect with conflict as a predictor	0.0008	0.0010	−0.0012, 0.0028
Indirect effect with closeness as a predictor	0.0000	0.0001	−0.0001, 0.0002
Total effect with closeness as a predictor	0.0023	0.0021	−0.0018, 0.0064

**Risk Adjustment**			
Discipline ➔ risk adjustment	**0.0089**⁎⁎	**0.0033**	**0.0025, 0.0155**
Conflict ➔ risk adjustment	0.0044	0.0032	−0.0018, 0.0107
Closeness ➔ risk adjustment	**0.0142**⁎	**0.0069**	**0.0004, 0.0280**
Risk adjustment ➔ self-harm	0.0052	0.0050	−0.0047, 0.0151
Discipline ➔ self-harm	0.0015	0.0012	−0.0009, 0.0038
Conflict ➔ self-harm	0.0008	0.0010	−0.0013, 0.0028
Closeness ➔ self-harm	0.0022	0.0021	−0.0019, 0.0064
Indirect effect with discipline as a predictor	0.0000	0.0000	−0.0000, 0.0001
Total effect with discipline as a predictor	0.0015	0.0012	−0.0008, 0.0038
Indirect effect with conflict as a predictor	0.0000	0.0000	−0.0000, 0.0001
Total effect with conflict as a predictor	0.0008	0.0010	−0.0012, 0.0028
Indirect effect with closeness as a predictor	0.0001	0.0001	−0.0001, 0.0002
Total effect with closeness as a predictor	0.0023	0.0021	−0.0018, 0.0064

**Quality of Decision Making**			
Discipline ➔ quality of decision making	0.0001	0.0007	−0.0012, 0.0014
Conflict ➔ quality of decision making	0.0009	0.0006	−0.0002, 0.0020
Closeness ➔ quality of decision making	0.0013	0.0013	−0.0014, 0.0041
Quality of decision making ➔ self-harm	0.0438	0.0340	0.0233, 0.1108
Discipline ➔ self-harm	0.0015	0.0012	−0.0009, 0.0038
Conflict ➔ self-harm	0.0008	0.0010	−0.0013, 0.0028
Closeness ➔ self-harm	0.0022	0.0021	−0.0019, 0.0064
Indirect effect with discipline as a predictor	0.0000	0.0000	−0.0001, 0.0001
Total effect with discipline as a predictor	0.0015	0.0012	−0.0008, 0.0038
Indirect effect with conflict as a predictor	0.0000	0.0000	−0.0000, 0.0001
Total effect with conflict as a predictor	0.0008	0.0010	−0.0012, 0.0028
Indirect effect with closeness as a predictor	0.0001	0.0001	−0.0001, 0.0002
Total effect with closeness as a predictor	0.0023	0.0021	−0.0018, 0.0064

**Deliberation Time**			
Discipline ➔ deliberation time	−8.5402	4.5849	−17.6100, 0.5297
Conflict ➔ deliberation time	−2.6458	4.0822	−10.7213, 0.5.4298
Closeness ➔ deliberation time	2.7076	9.7542	−16.7212, 22.0007
Deliberation time ➔ self-harm	0.0000	0.0000	0.0000, 0.0000
Discipline ➔ self-harm	0.0015	0.0012	−0.0009, 0.0038
Conflict ➔ self-harm	0.0008	0.0010	−0.0013, 0.0028
Closeness ➔ self-harm	0.0022	0.0021	−0.0019, 0.0064
Indirect effect with discipline as a predictor	−0.0000	0.0000	−0.0001, 0.0000
Total effect with discipline as a predictor	0.0015	0.0012	−0.0009, 0.0038
Indirect effect with conflict as a predictor	−0.0000	0.0000	−0.0000, 0.0000
Total effect with conflict as a predictor	0.0007	0.0010	−0.0013, 0.0028
Indirect effect with closeness as a predictor	0.0000	0.0000	−0.0000, 0.0001
Total effect with closeness as a predictor	0.0022	0.0021	−0.0019, 0.0064

**Total Time**			
Discipline ➔ total time	−0.0617	0.0347	−0.1302, 0.0068
Conflict ➔ total time	0.0320	0.0317	−0.0319, 0.0959
Closeness ➔ total time	0.0099	0.0786	−0.1471, 0.1669
Total time ➔ self-harm	−0.0000	0.0002	−0.0005, 0.0004
Discipline ➔ self-harm	0.0015	0.0012	−0.0009, 0.0038
Conflict ➔ self-harm	0.0008	0.0010	−0.0013, 0.0028
Closeness ➔ self-harm	0.0022	0.0021	−0.0019, 0.0064
Indirect effect with discipline as a predictor	0.0000	0.0000	−0.0000, 0.0000
Total effect with discipline as a predictor	0.0015	0.0012	−0.0008, 0.0038
Indirect effect with conflict as a predictor	−0.0000	0.0000	−0.0000, 0.0000
Total effect with conflict as a predictor	0.0007	0.0010	−0.0013, 0.0028
Indirect effect with closeness as a predictor	−0.0000	0.0000	−0.0000, 0.0000
Total effect with closeness as a predictor	0.0022	0.0021	−0.0018, 0.0064

**Delay Aversion**			
Discipline ➔ delay aversion	0.0010	0.0008	−0.0006, 0.0026
Conflict ➔ delay aversion	−0.0008	0.0007	−0.0023, 0.0007
Closeness ➔ delay aversion	−0.0015	0.0017	−0.0051, 0.0020
Delay aversion ➔ self-harm	**0.0624**⁎⁎⁎	**0.0193**	**0.0243, 0.1005**
Discipline ➔ self-harm	0.0015	0.0012	−0.0009, 0.0038
Conflict ➔ self-harm	0.0008	0.0010	−0.0013, 0.0028
Closeness ➔ self-harm	0.0022	0.0021	−0.0019, 0.0064
Indirect effect with discipline as a predictor	0.0001	0.0001	−0.0000, 0.0002
Total effect with discipline as a predictor	0.0015	0.0011	−0.0008, 0.0039
Indirect effect with conflict as a predictor	−0.0001	0.0001	−0.0001, 0.0000
Total effect with conflict as a predictor	0.0007	0.0010	−0.0013, 0.0028
Indirect effect with closeness as a predictor	−0.0001	0.0001	−0.0003, 0.0001
Total effect with closeness as a predictor	0.0021	0.0021	−0.0019, 0.0063

*Note*. B = unstandardised regression coefficient, SE = standard error, CI = confidence interval, CGT = Cambridge gambling task.

Adjusted for sex, ethnicity, birthweight, family structure, siblings, poverty, puberty, parent education, parent mental health, and parent smoking status.

⁎ p < .05.

⁎⁎ p < .01.

⁎⁎⁎ p < .001.

The correlations between the variables in the models can be found in supplementary file Tables 4 and 5, and were low to moderate. As expected, emotion regulation variables measured at age 3, 5 and 7 were highly correlated, and emotional dysregulation and independence & self-regulation were inversely related.

### 3.2 Models

#### 3.2.1 Direct effects

In model 1, the intercept of independence & self-regulation and the slope of emotional dysregulation were positively associated with self-harm (Table 2). In addition, delay aversion and conflict were positively associated with self-harm, whereas risk taking was negatively associated with self-harm (Model 1). There was evidence of a positive association between the slope of emotional dysregulation and self-harm, as well as between delay aversion and self-harm, even after adjustment for the covariates and confounders (Model 2) (Table 2). However, the intercept of independence & self-regulation, risk-taking and conflict were no longer significantly associated with self-harm after adjustment (Model 2). Finally, there were significant positive associations between self-harm and sex (female), ethnicity (white), parent mental health, and parent smoking status (smoker).

#### 3.2.2 Indirect and total effects

We tested the effects of early parenting on self-harm via emotion regulation and decision-making. In model 1, we found that conflict and closeness had indirect effects mediated by the slope of emotional dysregulation and the intercept of independence & self-regulation. Discipline had an indirect effect on self-harm via risk-taking (see supplementary file, Tables 6 and 7, for the direct, indirect and total effects in the unadjusted model with imputed data). In model 2 (Table 4), conflict and closeness continued to have indirect effects on self-harm via the slope of emotional dysregulation. However, they no longer had significant effects on self-harm via independence & self-regulation, and discipline no longer had a significant effect via risk-taking, after adjustment. Table 3, Table 4 present the results (direct, indirect and total effects) of our mediation models after adjustment in the imputed cases.

**Table 4:** Mediation (by emotion regulation) of parenting on self-harm in imputed cases.⁎

Direct and indirect paths	Imputed cases (n = 11,145)		
	B	SE	95 % CI
Slope of Emotional Dysregulation			
Discipline ➔ slope	0.0001	0.0002	−0.0002, 0.0004
Conflict➔ slope	**0.0009**⁎⁎⁎	**0.0001**	**0.0006, 0.0012**
Closeness ➔ slope	**−0.0013**⁎⁎⁎	**0.0003**	**−0.0019, 0.0007**
Slope ➔ self-harm	**0.3974**⁎⁎⁎	**0.0787**	**0.2426, 0.5522**
Discipline ➔ self-harm	0.0015	0.0012	−0.0009, 0.0038
Conflict ➔ self-harm	0.0008	0.0010	−0.0013, 0.0028
Closeness ➔ self-harm	0.0022	0.0021	−0.0019, 0.0064
Indirect effect with discipline as a predictor	0.0000	0.0001	−0.0001, 0.0002
Total effect with discipline as a predictor	0.0015	0.0012	−0.0008, 0.0038
Indirect effect with conflict as a predictor	**0.0004**⁎⁎⁎	**0.0001**	**0.0002, 0.0005**
Total effect with conflict as a predictor	0.0011	0.0010	−0.0009, 0.0031
Indirect effect with closeness as a predictor	**−0.0005**⁎⁎⁎	**0.0002**	**−0.0007, 0.0003**
Total effect with closeness as a predictor	0.0017	0.0021	−0.0024, 0.0058

Intercept of Emotional Dysregulation			
Discipline ➔ intercept	**0.0054**⁎⁎⁎	**0.0008**	**0.0039, 0.0069**
Conflict ➔ intercept	**0.0228**⁎⁎⁎	**0.0006**	**0.0216, 0.0240**
Closeness ➔ intercept	**−0.0118**⁎⁎⁎	**0.0017**	**−0.0151, −0.0085**
Intercept ➔ self-harm	0.0148	0.0208	−0.0261, 0.0557
Discipline ➔ self-harm	0.0015	0.0012	−0.0009, 0.0038
Conflict ➔ self-harm	0.0008	0.0010	−0.0013, 0.0028
Closeness ➔ self-harm	0.0022	0.0021	−0.0019, 0.0064
Indirect effect with discipline as a predictor	0.0001	0.0001	−0.0001, 0.0003
Total effect with discipline as a predictor	0.0016	0.0012	−0.0007, 0.0038
Indirect effect with conflict as a predictor	0.0003	0.0005	0.0002, 0.0005
Total effect with conflict as a predictor	0.0011	0.0009	−0.0007, 0.0029
Indirect effect with closeness as a predictor	−0.0002	0.0003	−0.0007, 0.0003
Total effect with closeness as a predictor	0.0021	0.0021	−0.0020, 0.0061

Slope of Independence & Self-regulation			
Discipline ➔ slope	**−0.0005**⁎⁎	**0.0002**	**−0.0008, −0.0002**
Conflict➔ slope	**−0.0005**⁎⁎⁎	**0.0001**	**−0.0007, −0.0002**
Closeness ➔ slope	0.0003	0.0003	−0.0003, 0.0008
Slope ➔ self-harm	−0.0745	0.0880	−0.2475, 0.0985
Discipline ➔ self-harm	0.0015	0.0012	−0.0009, 0.0038
Conflict ➔ self-harm	0.0008	0.0010	−0.0013, 0.0028
Closeness ➔ self-harm	0.0022	0.0021	−0.0019, 0.0064
Indirect effect with discipline as a predictor	0.0000	0.0000	−0.0001, 0.0001
Total effect with discipline as a predictor	0.0016	0.0012	−0.0007, 0.0038
Indirect effect with conflict as a predictor	0.0001	0.0001	−0.0001, 0.0001
Total effect with conflict as a predictor	0.0008	0.0010	−0.0012, 0.0028
Indirect effect with closeness as a predictor	0.0000	0.0000	−0.0001, 0.0000
Total effect with closeness as a predictor	0.0022	0.0021	−0.0019, 0.0063

Intercept of Independence & Self-regulation			
Discipline ➔ intercept	**−0.0013**⁎⁎	**0.0005**	**−0.0022, 0.0003**
Conflict ➔ intercept	**−0.0036**⁎⁎⁎	**0.0005**	**−0.0045, −0.0027**
Closeness ➔ intercept	**0.0133**⁎⁎⁎	**0.0010**	**0.0112, 0.0153**
Intercept ➔ self-harm	0.0159	0.0294	−0.0419, 0.0737
Discipline ➔ self-harm	0.0015	0.0012	−0.0009, 0.0038
Conflict ➔ self-harm	0.0008	0.0010	−0.0013, 0.0028
Closeness ➔ self-harm	0.0022	0.0021	−0.0019, 0.0064
Indirect effect with discipline as a predictor	−0.0000	0.0000	−0.0001, 0.0001
Total effect with discipline as a predictor	0.0015	0.0012	−0.0008, 0.0038
Indirect effect with conflict as a predictor	−0.0001	0.0001	−0.0003, 0.0002
Total effect with conflict as a predictor	0.0007	0.0010	−0.0013, 0.0027
Indirect effect with closeness as a predictor	−0.0002	0.0004	−0.0006, 0.001
Total effect with closeness as a predictor	0.0025	0.0021	−0.0016, 0.0065

*Note*. B = unstandardised regression coefficient, SE = standard error, CI = confidence interval, CGT = Cambridge gambling task.

Adjusted for sex, ethnicity, birthweight, family structure, siblings, poverty, parent education, parent mental health, and parent smoking status.

⁎ p < .05.

⁎⁎ p < .01.

⁎⁎⁎ p < .001.

### 3.3 Supplementary analysis

We conducted additional analyses (complete and imputed cases) including depression at age 14 as a confounding variable (see supplementary file, Table 8). We found that the positive association between the slope of emotional dysregulation and self-harm, and delay aversion and self-harm, were robust to adjustment.

## 4 Discussion

The present study aimed to test the relationship between early parenting and self-harm in adolescence, and explore mediation by emotion regulation trajectories and decision-making in childhood. In accordance with previous research, we found in a large, general population sample (n = 11,145) that 14.7 % (*n* = 1634) of 14-year olds had self-harmed in the past 12-months (Madge et al., 2008; Stallard et al., 2013). Furthermore, we found that chronic emotional dysregulation in childhood and delay aversion in late childhood were associated with self-harm in adolescence, and that early parent-child conflict and closeness had indirect effects on self-harm via chronic emotional dysregulation in childhood.

### 4.1 Emotional dysregulation

Our findings support that poor emotion regulation in childhood is a risk factor for self-harm in adolescence (Srinivasan et al., 2023; Wolff et al., 2019). Emotional dysregulation can be defined as an inability to manage emotional responses to stimuli, characterised by heightened emotional reactivity and maladaptive emotion regulation strategies that interfere with goal-directed activities (Thompson, 2019). A recent meta-analysis (Wolff et al., 2019) highlighted that individuals who engage in self-harm in adolescence experience greater emotional dysregulation, particularly with respect to heightened emotional reactivity and poor emotion regulation strategies. Thus, as well as acting as a coping strategy to manage negative emotions (Klonsky, 2007), self-harm may more broadly represent an individual's attempt to deal with underlying emotional dysregulation (Nock, 2009; Nock, 2010; Nock and Prinstein, 2005; Nock et al., 2008). The present findings indicate that children exhibiting emotional dysregulation that does not improve across early to middle childhood would benefit from interventions that teach them to recognise their feelings and promote effective strategies to manage emotion, as this may reduce the risk of self-harm in adolescence.

### 4.2 Parenting

To our knowledge this is the first longitudinal study to establish an indirect relationship between early parenting and self-harm in adolescence via chronic emotional dysregulation in childhood. This aligns with theories and cross-sectional evidence that advocate that early parenting contributes to emotional dysregulation, which in turn increases the risk of self-harm in adolescence (Adrian et al., 2011; Linehan, 1993). Our findings recommend that early parent-child relationships characterised by high conflict and low closeness are associated with poor emotion regulation that does not improve across childhood, which is associated with an increased likelihood of engaging in self-harm in adolescence. Previous research has emphasised the fundamental role that parents play in the socialisation of emotion (Eisenberg et al., 1998); modelling how to manage emotions and supporting their children to recognise and understand their feelings, until emotion regulation is internalised. (Buckholdt et al., 2009) examined the association between retrospective reports of parent socialisation of emotion and self-harm and found that parental punishment and neglect of expressions of sadness was related to self-harm. This relationship was mediated by negative evaluations of emotional experiences and a belief that nothing could be done to effectively manage emotion. In addition, the self-punishment hypothesis of self-harm contends that an early parenting environment that contributes to the belief that experiencing or expressing negative emotions should be punished results in an association between punishment and emotional relief. According to this theory, when an individual experiences negative emotion and is not punished by their parents, they feel a sense of cognitive dissonance that causes them discomfort. Therefore, self-harm is adopted as a form of self-punishment that gives the individual a sense of control and reaffirms their expectations (Chapman et al., 2006; Nock and Cha, 2009). Consequently, self-harm may also relieve distress by reducing interpersonal conflict and threat of external punishment, in addition to avoiding negative emotion (Chapman et al., 2006). Thus, it is plausible that parent-child relationships that are high in conflict and low in closeness do not provide the necessary environmental conditions, opportunities or tools for children to learn how to effectively manage and regulate their emotions, and that children with concomitant chronic emotional dysregulation are more likely to self-harm. Moving forward, family-based interventions that focus on improving the parent-child relationship (e.g., reducing conflict and increasing closeness) may help to reduce the risk of self-harm in adolescence.

### 4.3 Decision-making and reward processing

The finding that delay aversion is associated with self-harm in adolescence is consistent with literature suggesting altered decision-making and reward processing in individuals who self-harm. Furthermore, it provides direct evidence of a link between behavioural impulsivity and self-harm, while adding further distinction by highlighting that behavioural impulsivity is an antecedent to self-harm. Delay aversion is theoretically linked to impulsivity and can be defined as an intolerance to waiting to receive a reward, whereby an individual would rather receive a smaller, immediate than a larger, delayed reward (Sonuga-Barke et al., 2003). However, it is important to note that delay aversion measured by the CGT does not distinguish between the impulsive drive for immediate reward and the need to escape delay (Deakin et al., 2004). Nevertheless, recent conceptualisations recommend that delay aversion is distinct from inhibitory control and represents an individual's attempt to avoid the negative effects of delay rather than gain a more immediate reward (Sonuga-Barke et al., 2003). Thus, it is perhaps not surprising that we found that delay aversion in childhood is associated with self-harm in adolescence, if one considers that individuals may adopt self-harm as a strategy to escape and regulate negative emotions (Chapman et al., 2006; Nock, 2009; Nock, 2010). Overall, our findings suggest that adolescents who self-harm show decision-making biases in late childhood that align with the theory that individuals discount the long-term implications of self-harm (e.g., scarring) in favour of the short-term benefits (e.g., relief from negative emotion) (Tice et al., 2001). It has been posited that individuals engage in self-harm because it is more convenient, immediate and less effortful than other emotion regulation strategies (Nock and Cha, 2009). Previous research has shown that lack of premeditation (the inability to delay action in order to plan) and negative urgency (the tendency to act rashly in response to negative emotion) are associated with the development of self-harm, and lack of perseverance (the inability to persist with strategies) with the maintenance of self-harm (Glenn and Klonsky, 2013). Hence, while emotional dysregulation may evoke the desire to engage in self-harm, delay aversion may increase the likelihood of self-harm behaviour by limiting the individual's capacity to implement alternative emotion regulation strategies.

While the negative association between risk-taking and self-harm was not robust to adjustment, reduced risk-taking in individuals who self-harm would suggest greater sensitivity to punishment than reward. (Nock and Prinstein, 2004) propose that self-harm is maintained by reinforcement processes that differ across two dimensions: whether the consequences are intrapersonal or interpersonal, and whether reinforcement is positive or negative. Together, the present results provide some evidence that altered decision-making contributes to the negative intrapersonal (e.g., to avoid negative emotion) and interpersonal (e.g., to avoid punishment) reinforcement mechanisms of self-harm. It may be that temporary relief from negative feelings motivates impulsive behaviours that are biased to short-term gain to the detriment of long-term goals, with successful alleviation of distress and avoiding punishment leading to the negative reinforcement of self-harm behaviour. The experimental avoidance model (Chapman et al., 2006) suggests that individuals who self-harm have strong avoidance tendencies, stemming from deficits in emotion regulation and difficulties implementing effective coping strategies, and assumes that individuals engage in self-harm in order to avoid or escape uncomfortable internal experiences or the external conditions that elicit them (Chapman et al., 2011). As such, self-harm is likely maintained and strengthened by both escape conditioning and negative reinforcement. Individuals who demonstrate reduced risk-taking and greater delay aversion may be more likely to choose destructive coping strategies that have an immediate effect on negative emotions, rather than more adaptive strategies that take longer to alleviate distress, in order to avoid the discomfort of delay and external punishment. Overall, the present findings champion intervention strategies that explore underlying decision-making biases and help individuals to identify and address the thought processes that underpin their self-harming behaviour.

### 4.4 Limitations and future directions

Firstly, we must acquiesce that the present study design does not allow us to determine causality. That said, the longitudinal design allows us to determine the temporal association between risk factors (e.g., emotional dysregulation and poor decision-making arise prior to the onset of self-harm in adolescence). Secondly, self-harm was measured by a single, binary item at age 14 that examined whether the individuals had self-harmed in the last 12 months, which precluded us from assessing the specific self-harm behaviours that were present in the sample or ascertaining whether individuals had self-harmed earlier in their lifetime. Thirdly, while we recognised that self-report measures of parenting are vulnerable to several biases, the present study may have some additional benefit over retrospective reports of parenting by prospectively assessing parenting behaviours in early childhood. Finally, this study examined mediation through emotion regulation and decision-making independently, but it may be that the two interact to maximise the risk of self-harm in adolescence (Beauchaine et al., 2019). Therefore, it will be important to elucidate the precise relationships between parenting, emotion regulation, decision-making and reward processing in childhood and self-harm in adolescence.

### 4.5 Conclusion

Our findings indicate that early parenting (closeness and conflict) is associated with self-harm in adolescence through chronic emotional dysregulation in childhood, and that self-harm in adolescence is associated with delay aversion in late childhood. As such, there may be benefits to early identification and interventions for children exhibiting chronic emotional dysregulation and decision-making characterised by a bias for smaller, immediate over larger, delayed rewards. These findings, from a large, general population sample, provide an additional step towards understanding the psychopathogenesis of self-harm in adolescence.

## CRediT authorship contribution statement

EDL was involved in the conceptualisation of the research project, data analysis, and writing the manuscript. EF acted as EDL's supervisor and assisted with conceptualisation, data analysis and editing the manuscript. The authors read and approved the final manuscript.

## Declaration of competing interest

None.

## Appendix A Supplementary data

Supplementary material Image 1
