# Supplementary material for: Parenting in the early years and self-harm in adolescence: The role of control and reward systems in childhood
Source: J Affect Disord. 2023 Oct 15;339:788–98. doi: 10.1016/j.jad.2023.07.061 (PMC11139656; doi:10.1016/j.jad.2023.07.061)
Supplement: Supplementary file 1 — Supplementary material [file mmc1.docx]

Supplementary File

Sex was coded as a binary variable at 9-months: male (0), female (1). Ethnicity was initially coded as White (1), Mixed (2), Indian (3), Pakistani and Bangladeshi (4), Black or Black British (5) other Ethnic Group (6) at 9-months in line with the UK census categorisation of ethnicity, but was recoded into a binary variable: non-white (0), white (1). Family structure was coded as a binary variable at 9-months: one-parent (0), two-parent (1). Poverty was coded as a binary variable based on whether the family income was above or below the median 60% poverty indicator at 9-months: Above (1), Below (0). Higher education was recoded into a binary variable based on whether the main caregiver had completed higher education or equivalent NVQ level (4+) qualification at 9-months: no (0), yes (1). Smoking status was coded as a binary variable at 9-months: no (0), yes (1). Finally, pubertal status was coded into a binary variable based on whether the individual was showing any evidence of puberty at age 11: no (0), yes (1).

Table 1.

Descriptive Statistics in the Analytic Sample (Unweighted Data)

|  |  |  |  | Self-Harm | | | | |  |
| --- | --- | --- | --- | --- | --- | --- | --- | --- | --- |
|  |  |  |  | Yes |  |  | No |  |  |
|  |  |  |  | N |  | % | N | % |  |
| *Age 9- months* |  |  |  |  |  |  |  |  |  |
| Sex |  |  |  |  |  |  |  |  |  |
|  |  | Female |  | 1148 |  | 73 | 4283 | 46.6 | X^2^_(1)_ = 373.724, |
|  |  | Male |  | 424 |  | 27 | 4899 | 53.4 | p<.001 |
| Ethnicity |  |  |  |  |  |  |  |  |  |
|  |  | White |  | 1382 |  | 88.1 | 7495 | 81.8 | X^2^_(1)_ =37.765, |
|  |  | Non-White |  | 186 |  | 11.9 | 1669 | 18.2 | p<.001 |
| *Household Factors* |  |  |  |  |  |  |  |  |  |
| Poverty (OECD 60% median income indicator) |  |  |  |  |  |  |  |  |  |
|  |  | Above |  | 1041 |  | 66.3 | 6249 | 68.7 | X^2^_(1)_ =3.551, |
|  |  | Below |  | 528 |  | 33.7 | 2862 | 31.3 | p=.060 |
| Family Structure |  |  |  |  |  |  |  |  |  |
|  |  | Two-parent |  | 1325 |  | 84.3 | 7964 | 86.7 | X^2^_(1)_ = 6.776, |
|  |  | One-parent |  | 247 |  | 15.7 | 1219 | 13.3 | p=.009 |
| Siblings |  |  |  |  |  |  |  |  |  |
|  |  | 0 |  | 647 |  | 41.2 | 3819 | 41.6 | X^2^_(3)_ |
|  |  | 1 |  | 564 |  | 35.9 | 3225 | 35.1 | =.812, |
|  |  | 2 |  | 243 |  | 15.5 | 1400 | 15.2 | p=.847 |
|  |  | 3+ |  | 118 |  | 7.5 | 739 | 8 |  |
| *Parent Factors* |  |  |  |  |  |  |  |  |  |
| Education (Higher Education, NVQ 4+) |  |  |  |  |  |  |  |  |  |
|  |  | Yes |  | 501 | 32.8 |  | 3201 | 36 | X^2^_(1)_ =5.768, |
|  |  | No |  | 1027 | 67.2 |  | 5698 | 64 | p=.016 |
| Smoking Status |  |  |  |  |  |  |  |  |  |
|  |  | Yes |  | 530 | 33.7 |  | 2294 | 25 | X^2^_(1)_ =52.775, |
|  |  | No |  | 1041 | 66.3 |  | 6880 | 75 | p<.001 |

|  |  |  |  | N | M (SE) | | N | | M (SE) | |  |  |
| --- | --- | --- | --- | --- | --- | --- | --- | --- | --- | --- | --- | --- |
| *Age 9- months* |  |  |  |  |  |  | |  | |  | | |
| Parent Mental Health |  |  |  | 1528 | 1.89 (.048) | 8811 | | 1.76 (.018) | | t(2002.64) = 5.387, p<.001 | | |
|  |  |  |  |  |  |  | |  | |  | | |
| *Age 3* |  |  |  |  |  |  | |  | |  | | |
| Closeness |  |  |  | 1318 | 33.68 (.062) | 7366 | | 33.63 (.025) | | t(8682) = .894, p=.371 | | |
| Conflict |  |  |  | 1342 | 17.61 (.165) | 7469 | | 16.95 (.067) | | t(8809) = 3.807, p<.001 | | |
| Discipline |  |  |  | 1249 | 2.588 (.141) | 8548 | | 2.615 (.004) | | t(8171) = 1.138, p=.260 | | |
| Independence & Self Regulations |  |  |  | 1471 | 2.485 (.004) | 8337 | | 2.485 (.004) | | t(9806) = 2.018, p=.044 | | |
| Emotional Dysregulation |  |  |  | 1471 | 1.882 (.012) | 8339 | | 1.863 (.005) | | t(9808) = 1.497, p=.027 | | |
|  |  |  |  |  |  |  | |  | |  | | |
| *Age 5* |  |  |  |  |  |  | |  | |  | | |
|  |  |  |  |  |  |  | |  | |  | | |
| Independence & Self Regulations |  |  |  | 1533 | 2.541 (.009) | 8697 | | 2.533 (.004) | | t(10228) =.867, p=.386 | | |
| Emotional Dysregulation |  |  |  | 1533 | 1.745 (.012) | 8697 | | 1.697 (.005) | | t(10228) = 3.781, p<.001 | | |
|  |  |  |  |  |  |  | |  | |  | | |
| *Age 7* |  |  |  |  |  |  | |  | |  | | |
| Independence & Self Regulations |  |  |  | 1487 | 2.514 (.368) | 8546 | | 2.517 (.361) | | t(10031) =-.377, p=.701 | | |
| Emotional Dysregulation |  |  |  | 1487 | 1.757 (.487) | 8458 | | 1.694 (.466) | | t(10033) = 4.833, p<.001 | | |
| *Age 11* |  |  |  |  |  |  | |  | |  | | |
| Test Duration |  |  |  | 1495 | 9.97 (.110) | 8607 | | 9.94 (.156) | | t(10100) =.070, p=.944 | | |
| Deliberation Time |  |  |  | 1494 | 3397.11 (34.641) | 8601 | | 3306.92 (14.113) | | t(10093) = 2.450, p=.014 | | |
| Delay Aversion |  |  |  | 1489 | .290 (.006) | 8568 | | .283 (.003) | | t(10055) = .970, p=.332 | | |
| Quality of Decision Making |  |  |  | 1494 | .809 (.004) | 8601 | | .806 (.002) | | t(10093) =.682, p=.495 | | |
| Risk Adjustment |  |  |  | 1494 | .683 (.028) | 8600 | | .676 (.011) | | t(10092) = .247, p=.805 | | |
| Risk Taking |  |  |  | 1494 | .498 (.004) | 8600 | | .532 (.002) | | t(10092) = -7.362, p<.001 | | |

Table 2.

Bias Analysis for Categorical Variables in the Analytic and Non-Analytic Samples.

|  |  | Sample | | | | |
| --- | --- | --- | --- | --- | --- | --- |
|  |  | Non-Analytic |  | Analytic |  |  |
| Variable |  | N | % | N | % |  |
| Sex |  |  |  |  |  |  |
|  | Female | 3582 | 46 | 5431 | 50.5 | X^2^_(1)_ =36.454, |
|  | Male | 4203 | 54 | 5323 | 49.5 | p<.001 |
| Ethnicity |  |  |  |  |  |  |
|  | White | 6400 | 82.5 | 8877 | 82.7 | X^2^_(1)_ = .182 |
|  | Non-White | 1360 | 17.5 | 1855 | 17.3 | P=.670 |
| *Household Factors* |  |  |  |  |  |  |
| Poverty (OECD 60% median income indicator) |  |  |  |  |  |  |
|  | Above | 4267 | 55.2 | 7335 | 68.4 | X^2^_(1)_ = 335.967 |
|  | Below | 3466 | 44.8 | 3390 | 31.6 | P<.001 |
| Family structure |  |  |  |  |  |  |
|  | Two-parent  One-parent | 6057 | 77.8 | 9289 | 86.4 | X^2^_(1)_ = 232.365 |
|  |  | 1728 | 22.2 | 1466 | 13.6 | P<.001 |
| Siblings |  |  |  |  |  |  |
|  | 0 | 3227 | 41.5 | 4466 | 41.5 | X^2^_(3)_ =7.100 |
|  | 1 | 2644 | 34 | 3789 | 35.2 | p=.069 |
|  | 2 | 1223 | 15.7 | 1643 | 15.3 |  |
|  | 3+ | 691 | 8.9 | 857 | 8 |  |
| *Parent Factors* |  |  |  |  |  |  |
| Education (Higher Education e.g., NVQ 4+) |  |  |  |  |  |  |
|  | Yes | 1666 | 22.2 | 3702 | 35.5 | X^2^_(1)_ = 367.531, |
|  | No | 5835 | 77.8 | 6725 | 64.5 | p<.001 |
| Smoking Status |  |  |  |  |  |  |
|  | Yes | 2791 | 35.9 | 2824 | 26.3 | X^2^_(1)_ = 198.416 |
|  | No | 4971 | 64.1 | 7921 | 73.7 | p<.001 |

Table 3.

Bias Analysis for Continuous Variables in the Analytic and Non-Analytic Samples

|  | Sample |  |  |
| --- | --- | --- | --- |
|  | Non-Analytic  (8086) | Analytic  (11,145) |  |
|  | M (SE) | M (SE) | *t* (df)                  p |
| *Age 9-months* |  |  |  |
| Psychological Distress (Malaise) | 1.66 (.017) | 1.76 (.021) | t(15461.015) =3.486, p<.001 |
|  |  |  |  |
| *Age 3* |  |  |  |
| Closeness | 33.27 (.045) | 33.63 (.023) | t(6596.283) =-7.237, p<.001 |
| Conflict | 17.32 (.092) | 17.05 (.062) | t(8372.608) =2.439, p=.015 |
|  |  |  |  |
| Discipline | 20.11 (4.92) | 19.61 (5.18) | t(7546.264) =-4.969, p<.001 |
|  |  |  |  |
| Independence and Self-Regulation | 2.44 (.005) | 2.47 (.003) | t(14820) =-4.792 p<.001 |
| Emotional Dysregulation | 1.92 (.006) | 1.87 (.005) | t(14822) =6.704 p<.001 |
| *Age 5* |  |  |  |
| Independence and Self-regulation | 2.49 (.005) | 2.53 (.003) | t(7984.98) =5.942, p<.001 |
| Emotional Dysregulation | 1.79 (.007) | 1.70 (.004) | t(14761) =10.666, p<.001 |
| *Age 7* |  |  |  |
| Independence and Self-regulation | 2.47 (.007) | 2.52 (.004) | t(5606.44) =-6.980, p<.001 |
| Emotional Dysregulation | 1.80 (.008) | 1.70 (.005) | t(13477) =10.674, p<.001 |

*Age 11*

| Test Duration | 2.497 (.006) | 2.534 (.003) | t(7984.988) =-5.728, p<.001 |
| --- | --- | --- | --- |
| Deliberation Time | 1.793 (.007) | 1.704 (.005) | t(14761) =10.750, p<.001 |
| Delay Aversion | .306 (.005) | .284 (.003) | t(12445) =3.741, p<.001 |
| Quality of Decision Making | .779 (.004) | .807 (.002) | t(3582.065) =-7.188, p<.001 |
| Risk Adjustment | .513 (.021) | .677 (.010) | t(12510) =-7.021, p<.001 |
| Risk Taking | .541 (.004) | .527 (.002) | t(12510) =3.656, p<.001 |

Table 4.

Correlations of the model variables in the analytic sample (n=11,145)

| Variable | 1 | 2 | 3 | 4 | 5 | 6 | 7 | 8 | 9 | 10 | 11 | 12 | 13 | 14 |
| --- | --- | --- | --- | --- | --- | --- | --- | --- | --- | --- | --- | --- | --- | --- |
| 1.Sex | 1 |  |  |  |  |  |  |  |  |  |  |  |  |  |
| 2. Ethnicity | -.008 | 1 |  |  |  |  |  |  |  |  |  |  |  |  |
| 3. Birthweight | -.104** | .164** | 1 |  |  |  |  |  |  |  |  |  |  |  |
| 4. Family Structure | .002 | .035** | .069** | 1 |  |  |  |  |  |  |  |  |  |  |
| 5. Siblings | .005 | -.122** | .056** | .043** | 1 |  |  |  |  |  |  |  |  |  |
| 6. Poverty | .017 | -.256** | -.110** | -.427** | .208** | 1 |  |  |  |  |  |  |  |  |
| 7. Parent Education | -.017 | .077** | .079** | .203** | -.128** | -.352** | 1 |  |  |  |  |  |  |  |
| 8. Parent Mental Health | -.017 | -.062** | -.052** | -.098** | .061** | .151** | -.104** | 1 |  |  |  |  |  |  |
| 9. Smoking Status | -.006 | .163** | -.118** | -.252** | .032** | .229** | -.246** | .142** | 1 |  |  |  |  |  |
| 10. Conflict | -.039** | -.029** | -.035** | -.094** | -.081** | .098** | -.052** | .263** | .111** | 1 |  |  |  |  |
| 11. Closeness | .076** | .044** | .028* | .084** | -.045** | -.147** | .115** | -.115** | -.092** | -.269** | 1 |  |  |  |
| 12. Independence & Self-Regulation (3) | .094** | .062** | .028** | .006 | -.017 | -.035** | .043** | -.051** | -.005 | -.153** | .196** | 1 |  |  |
| 13. Emotional Dysregulation (3) | -.069** | .056** | -.043** | -.123** | -.021* | .185** | -.199** | .196** | .158** | .516** | -.198** | -.096** | 1 |  |
| 14. Independence & Self-Regulation (5) | .138** | -.089** | .050** | .033** | -.013 | -.071** | .065** | -.087** | -.050** | -.190** | .178** | .359** | -.175** | 1 |
| 15. Emotional Dysregulation (7) | -.080** | .061** | -.075** | -.128** | -.009 | .197** | -.186** | .218** | .144** | .440** | -.197** | -.120** | .526** | -.250** |
| 16. Independence & Self-Regulation (7) | .127** | -.082** | .050** | .029** | -.013 | -.074** | .079** | -.090** | -.048** | -.183** | .155** | .304** | -.182** | .479** |
| 17. Emotional Dysregulation (7) | -.095** | -.003 | -.065** | -.127** | -.024* | .198** | -.183** | .215** | .136** | .398** | -.194** | -.110** | .469** | -.224** |
| 18. Test Duration | .017 | -.012 | .010 | .011 | .003 | -.002 | .020 | -.005 | .006 | .004 | .005 | .000 | .004 | .000 |
| 19. Delay Aversion | -.110** | .021* | -.014 | -.044* | -.015 | .017 | -.027** | .018 | .033** | .038** | -.033** | -.011 | .057** | -.025* |
| 20. Deliberation Time | .046** | -.116** | -.011 | -.015 | .001 | .015 | -.041** | .032** | .017 | -.006 | .006 | .004 | .022* | -.002 |
| 21. Overall Proportional Bet | -.254** | .022* | .003 | -.027** | .010 | .057** | -.042** | .034** | .008 | .059** | -.056** | -.053** | .074** | -.76** |
| 22. Quality Decision Making | .012 | .073** | .029** | .088** | -.054** | -.111** | .121** | -.041** | -.079** | -.021 | .028* | .005 | -.067** | .026* |
| 23. Risk Adjustment | -.019 | -.100** | .045** | .067** | -.033** | -.118** | .127** | -.038** | -.053** | -.027* | .041** | .006 | -.069** | .038** |
| 24. Risk Taking | -.275** | .065** | .011 | -.024* | .004 | .040** | -.031** | .036** | .008 | .056** | -.049** | -.053** | .066** | -.073** |
| 25. Discipline | -.102** | .028** | .029* | .013 | -.129** | -.053** | .034** | .125** | .019 | .431** | -.065** | -.062** | .309** | -.113** |
| 26. Pubertal status | .223** | .059** | -.011 | -.048** | -.076** | .018 | -.042** | .026* | .057** | .021 | .020 | .033** | .027** | .053** |
| 27. Self-Harm | .186** |  | -.014 | -.025** | -.002 | .018 | -.024* | .056** | .070** | .041** | .010 | .020* | .015 | .009 |

***Note.*** 1-9; covariates at 9-months (0= male, non-white, one parent family, below median income, no higher education, non-smoker; 1=female, white, two parent family, above median income, higher education, smoker), 10-11; parent-child relationship scale at age 3, 12-17; Child Social Behaviour Questionnaire at ages 3, 5 and 7, 18-24; Cambridge Gambling Task at age 11, 25; parent discipline at age 3, 26; pubertal status at age 11 (0=no: 1=yes), 27; self-harm at age 14 (0=no: 1= yes).

Table 5.

Correlations of the model variables in the analytic sample (n=11,145)

| Variable | 15 | 16 | 17 | 18 | 19 | 20 | 21 | 22 | 23 | 24 | 25 | 26 | 27 |
| --- | --- | --- | --- | --- | --- | --- | --- | --- | --- | --- | --- | --- | --- |
| 1. Sex |  |  |  |  |  |  |  |  |  |  |  |  |  |
| 2. Ethnicity |  |  |  |  |  |  |  |  |  |  |  |  |  |
| 3. Birthweight |  |  |  |  |  |  |  |  |  |  |  |  |  |
| 4. Family Structure |  |  |  |  |  |  |  |  |  |  |  |  |  |
| 5. Siblings |  |  |  |  |  |  |  |  |  |  |  |  |  |
| 6. Poverty |  |  |  |  |  |  |  |  |  |  |  |  |  |
| 7. Parent Education |  |  |  |  |  |  |  |  |  |  |  |  |  |
| 8. Parent Mental Health |  |  |  |  |  |  |  |  |  |  |  |  |  |
| 9. Smoking Status |  |  |  |  |  |  |  |  |  |  |  |  |  |
| 10. Conflict |  |  |  |  |  |  |  |  |  |  |  |  |  |
| 11. Closeness |  |  |  |  |  |  |  |  |  |  |  |  |  |
| 12. Independence & Self-Regulation |  |  |  |  |  |  |  |  |  |  |  |  |  |
| 13. Emotional Dysregulation |  |  |  |  |  |  |  |  |  |  |  |  |  |
| 14. Independence & Self-Regulation |  |  |  |  |  |  |  |  |  |  |  |  |  |
| 15. Emotional Dysregulation | 1 |  |  |  |  |  |  |  |  |  |  |  |  |
| 16. Independence & Self-Regulation | -.261** | 1 |  |  |  |  |  |  |  |  |  |  |  |
| 17. Emotional Dysregulation | .642** | -.319** | 1 |  |  |  |  |  |  |  |  |  |  |
| 18. Test Duration | -.006 | .000 | .003 | 1 |  |  |  |  |  |  |  |  |  |
| 19. Delay Aversion | .053** | -.045** | .073** | -.124** | 1 |  |  |  |  |  |  |  |  |
| 20. Deliberation Time | .029** | -.040** | .022* | .082** | -.158** | 1 |  |  |  |  |  |  |  |
| 21. Overall Proportional Bet | .089** | -.089* | .090** | -.025* | .167** | -.073** | 1 |  |  |  |  |  |  |
| 22. Quality Decision Making | -.067** | .053** | -.084** | -.014 | -.057** | -.211** | .132** | 1 |  |  |  |  |  |
| 23. Risk Adjustment | -.080** | .054** | -.088** | .024* | -.151** | -.052** | -.223** | .280** |  |  |  |  |  |
| 24. Risk Taking | .082** | -.080** | .085** | -.020* | .192** | -.061** | .963** | .103** | -.182** |  |  |  |  |
| 25. Discipline | .259** | -.118** | .221** | -.011 | .047** | -.023* | .068** | -.014 | .004 | .074** |  |  |  |
| 26. Pubertal Status | .008 | .033** | .012 | .015 | -.043** | .020* | -.068** | .004 | -.001 | -.075** | -.008 |  |  |
| 27. Self-Harm | .037** | -.004 | .048** | .001 | .010 | .024* | -.073** | .007 | .002 | -.073** | .013 | .068** |  |

***Note.*** 1-9; covariates at 9-months (0= male, non-white, one parent family, below median income, no higher education, non-smoker: 1=female, white, two parent family, above median income, higher education, smoker), 10-11; parent-child relationship scale at age 3, 12-17; Child Social Behaviour Questionnaire at ages 3, 5 and 7, 18-24; Cambridge Gambling Task at age 11, 25; parent discipline at age 3, 26; pubertal status at age 11 (0=no: 1=yes), 27; self-harm at age 14 (0=no: 1=yes).

Table 6.

Mediation (by CGT) of Parenting on Self-Harm (Unadjusted)

| **Direct and Indirect Paths** | | **Imputed Cases (n=11,145)** | | |
| --- | --- | --- | --- | --- |
|  |  | B | SE | 95% CI |
| **Risk Taking** | | | | |
| Discipline 🡪 Risk Taking |  | **.0020***** | **.0005** | **.0009, .0030** |
| Conflict 🡪 Risk Taking |  | -.0081 | .0006 | -.0019, .0003 |
| Closeness 🡪 Risk Taking |  | -.0009 | .0012 | -.0033, .0015 |
| Risk Taking 🡪 Self-Harm |  | **-.1767***** | **.0299** | **-.2356,.-.1178** |
| Discipline 🡪 Self-Harm |  | .0003 | .0012 | -.0020, .0026 |
| Conflict 🡪 Self-Harm |  | **.0023***** | **.0010** | **.0024, .0043** |
| Closeness 🡪 Self-Harm |  | .0026 | .0022 | -.0018, .0069 |
| Indirect effect with Discipline as a predictor |  | **-.0004***** | **.0001** | **-.0006, -.0001** |
| Total effect with Discipline as a predictor |  | -.0001 | .0012 | -.0024, .0022 |
| Indirect effect with Conflict as a predictor |  | .0002 | .0001 | -.0001, .0003 |
| Total effect with Conflict as a predictor |  | **.0024*** | **.0010** | **.0004, .0044** |
| Indirect effect with Closeness as a predictor |  | .0002 | .0002 | -.0003, .0006 |
| Total effect with Closeness as a predictor |  | .0027 | .0022 | -.0016, .0071 |
| **Risk Adjustment** | | | | |
| Discipline 🡪 Risk Adjustment |  | **.0089** | **.0033** | **.0025, .0155** |
| Conflict 🡪 Risk Adjustment |  | .0044 | .0032 | -.0018, .0107 |
| Closeness 🡪 Risk Adjustment |  | **.0142*** | **.0069** | **.0004, .0281** |
| Risk Adjustment 🡪 Self-Harm |  | -.0023 | .0051 | -.0124, .0078 |
| Discipline 🡪 Self-Harm |  | .0003 | .0012 | -.0020, .0026 |
| Conflict 🡪 Self-Harm |  | **.0023***** | **.0010** | **.0024, .0043** |
| Closeness 🡪 Self-Harm |  | .0026 | .0022 | -.0018, .0069 |
| Indirect effect with Discipline as a predictor |  | -.0000 | .0000 | -.0001, .0001 |
| Total effect with Discipline as a predictor |  | .0003 | .0012 | -.0020, .0026 |
| Indirect effect with Conflict as a predictor |  | -.0000 | .0000 | -.0001, .0000 |
| Total effect with Conflict as a predictor |  | **.0023*** | **.0020** | **.0002, .0043** |
| Indirect effect with Closeness as a predictor |  | -.0000 | .0001 | -.0002, .0001 |
| Total effect with Closeness as a predictor |  | .0025 | .0022 | -.0018, .0069 |
| **Quality of Decision Making** | | | | |
| Discipline 🡪 Quality of Decision Making |  | .0001 | .0007 | -.0012, .0014 |
| Conflict 🡪 Quality of Decision Making |  | .0009 | .0006 | -.0002, .0020 |
| Closeness 🡪 Quality of Decision Making |  | .0014 | .0014 | -.0012, .0041 |
| Quality of Decision Making 🡪 Self-Harm |  | .0673 | .0196 | .0135, .0906 |
| Discipline 🡪 Self-Harm |  | .0003 | .0012 | -.0020, .0026 |
| Conflict 🡪 Self-Harm |  | **.0023***** | **.0010** | **.0024, .0043** |
| Closeness 🡪 Self-Harm |  | .0026 | .0022 | -.0018, .0069 |
| Indirect effect with Discipline as a predictor |  | .0000 | .0000 | -.0001, .0001 |
| Total effect with Discipline as a predictor |  | .0003 | .0012 | -.0020, .0026 |
| Indirect effect with Conflict as a predictor |  | .0000 | .0000 | -.0000, .0001 |
| Total effect with Conflict as a predictor |  | **.0023*** | **.0010** | **.0003, .0043** |
| Indirect effect with Closeness as a predictor |  | .0001 | .0001 | -.0001, .0003 |
| Total effect with Closeness as a predictor |  | .0026 | .0022 | -.0017, .0069 |
| **Deliberation Time** | | | | |
| Discipline 🡪 Deliberation Time |  | -8.6138 | 4.5780 | -17.6739, .4464 |
| Conflict 🡪 Deliberation Time |  | -2.5184 | 4.0723 | -10.5756, 5.5388 |
| Closeness 🡪 Deliberation Time |  | 2.8794 | 9.7390 | -16.3835, 22.1422 |
| Deliberation Time 🡪 Self-Harm |  | 5.22 | 3.60 | -1.87, .0000 |
| Discipline 🡪 Self-Harm |  | .0003 | .0012 | -.0020, .0026 |
| Conflict 🡪 Self-Harm |  | **.0023***** | **.0010** | **.0024, .0043** |
| Closeness 🡪 Self-Harm |  | .0026 | .0022 | -.0018, .0069 |
| Indirect effect with Discipline as a predictor |  | -.0000 | .0000 | -.0001, .0000 |
| Total effect with Discipline as a predictor |  | .0003 | .0012 | -.0020, .0026 |
| Indirect effect with Conflict as a predictor |  | -.000 | .0000 | -.0001, .0000 |
| Total effect with Conflict as a predictor |  | **.0023*** | **.0010** | **.0003, .0043** |
| Indirect effect with Closeness as a predictor |  | .0000 | .0001 | -.0001, .0001 |
| Total effect with Closeness as a predictor |  | .0026 | .0022 | -.0017, .0069 |
| **Total Time** | | | | |
| Discipline 🡪 Total Time |  | -.0623 | .0348 | -.1311, .0065 |
| Conflict 🡪 Total Time |  | .0330 | .0317 | -.0309, .1691 |
| Closeness 🡪 Total Time |  | .0111 | .0791 | -.1468, .1691 |
| Total Time 🡪 Self-Harm |  | .0001 | .0002 | -.0003, .0005 |
| Discipline 🡪 Self-Harm |  | .0003 | .0012 | -.0020, .0026 |
| Conflict 🡪 Self-Harm |  | **.0023***** | **.0010** | **.0024, .0043** |
| Closeness 🡪 Self-Harm |  | .0026 | .0022 | -.0018, .0069 |
| Indirect effect with Discipline as a predictor |  | -.0000 | .0000 | -.0000, .0000 |
| Total effect with Discipline as a predictor |  | .0003 | .0012 | -.0020, .0026S |
| Indirect effect with Conflict as a predictor |  | .0000 | .0000 | -.0000, .0000 |
| Total effect with Conflict as a predictor |  | **.0023*** | **.0010** | **.0003, .0043** |
| Indirect effect with Closeness as a predictor |  | .0000 | .0000 | -.0000, .0000 |
| Total effect with Closeness as a predictor |  | .0026 | .0022 | -.0017, .0069 |
| **Delay Aversion** | | | | |
| Discipline 🡪 Delay Aversion |  | .0010 | .0018 | -.0052, .0026 |
| Conflict 🡪 Delay Aversion |  | -.0009 | .0007 | -.0024, .0006 |
| Closeness 🡪 Delay Aversion |  | -.0016 | .0018 | -.0052, .0019 |
| Delay Aversion 🡪 Self-Harm |  | **.0520**** | **.0196** | **.0135, .0906** |
| Discipline 🡪 Self-Harm |  | .0003 | .0012 | -.0020, .0026 |
| Conflict 🡪 Self-Harm |  | **.0023***** | **.0010** | **.0024, .0043** |
| Closeness 🡪 Self-Harm |  | .0026 | .0022 | -.0018, .0069 |
| Indirect effect with Discipline as a predictor |  | .0001 | .0000 | -.0000, .0001 |
| Total effect with Discipline as a predictor |  | .0004 | .0012 | -.0019, .0027 |
| Indirect effect with Conflict as a predictor |  | -.0000 | .0000 | -.0001, .0000 |
| Total effect with Conflict as a predictor |  | **.0022*** | **.0010** | **.0002, .0042** |
| Indirect effect with Closeness as a predictor |  | -.0001 | .0001 | -.0003, .0001 |
| Total effect with Closeness as a predictor |  | .0025 | .0022 | -.0018, .0069 |
| **Note.** B=unstandardized regression coefficient, SE=standard error, CI=confidence interval, CGT=Cambridge gambling task  p<.05*, p<.01**, p<.001*** | | | | |

Table 7.

Mediation (by Emotion Regulation) of Parenting on Self-Harm (Unadjusted)

| **Direct and Indirect Paths** | | **Imputed Cases (n=11,145)** | | |
| --- | --- | --- | --- | --- |
|  |  | B | SE | 95% CI |
| **Emotional Dysregulation**  **Slope** | | | | |
| Discipline 🡪 Slope |  | .0001 | .0002 | -.0002, .0004 |
| Conflict🡪 Slope |  | **.0009***** | **.0001** | **.0006, .0012** |
| Closeness 🡪 Slope |  | **-.0013***** | **.0003** | **-.0019, -.0007** |
| Slope 🡪 Self-Harm |  | **.3908***** | **.0850** | **.2236, .5580** |
| Discipline 🡪 Self-Harm |  | .0003 | .0012 | -.0020, .0026 |
| Conflict 🡪 Self-Harm |  | **.0023***** | **.0010** | **.0024, .0043** |
| Closeness 🡪 Self-Harm |  | .0026 | .0022 | -.0018, .0069 |
| Indirect effect with Discipline as a predictor |  | .0000 | .0001 | -.0001, .0002 |
| Total effect with Discipline as a predictor |  | .0003 | .0012 | -.0019, .0026 |
| Indirect effect with Conflict as a predictor |  | **.0003***** | **.0001** | **.0002, .0005** |
| Total effect with Conflict as a predictor |  | **.0026*** | **.0010** | **.0006, .0046** |
| Indirect effect with Closeness as a predictor |  | **-.0005**** | **.0002** | **-.0008, -.0002** |
| Total effect with Closeness as a predictor |  | .0021 | .0022 | -.0022, .0064 |
| **Emotional Dysregulation**  **Intercept** | | | | |
| Discipline 🡪 Intercept |  | **.0054***** | **.0008** | **.0039, .0069** |
| Conflict 🡪 Intercept |  | **.0228***** | **.0006** | **.0216, .0240** |
| Closeness 🡪 Intercept |  | **-.0118***** | **.0017** | **-.0151, -.0085** |
| Intercept 🡪 Self-Harm |  | .0344 | .0201 | -.0052, .0739 |
| Discipline 🡪 Self-Harm |  | .0003 | .0012 | -.0020, .0026 |
| Conflict 🡪 Self-Harm |  | **.0023***** | **.0010** | **.0024, .0043** |
| Closeness 🡪 Self-Harm |  | .0026 | .0022 | -.0018, .0069 |
| Indirect effect with Discipline as a predictor |  | .0002 | .0002 | -.0000, .0004 |
| Total effect with Discipline as a predictor |  | .0005 | .0012 | -.0018, .0028 |
| Indirect effect with Conflict as a predictor |  | .0008 | .0000 | -.0001, .0000 |
| Total effect with Conflict as a predictor |  | **.0031***** | **.0009** | **.0012, .0049** |
| Indirect effect with Closeness as a predictor |  | -.0004 | .0002 | -.0009, .0001 |
| Total effect with Closeness as a predictor |  | .0021 | .0022 | -.0022, .0064 |
| **Independence and Self-Regulation**  **Slope** | | | | |
| Discipline 🡪 Slope |  | **-.0005**** | **.0002** | **-.0008, -.0002** |
| Conflict🡪 Slope |  | **-.0005***** | **.0001** | **-.0007, -.0002** |
| Closeness 🡪 Slope |  | .0003 | .0003 | -.0003, .0008 |
| Slope 🡪 Self-Harm |  | -.0156 | .0902 | -.1929, .1617 |
| Discipline 🡪 Self-Harm |  | .0003 | .0012 | -.0020, .0026 |
| Conflict 🡪 Self-Harm |  | **.0023***** | **.0010** | **.0024, .0043** |
| Closeness 🡪 Self-Harm |  | .0026 | .0022 | -.0018, .0069 |
| Indirect effect with Discipline as a predictor |  | .0000 | .0000 | -.0001, .0001 |
| Total effect with Discipline as a predictor |  | .0003 | .0012 | -.0019, .0026 |
| Indirect effect with Conflict as a predictor |  | .0000 | .0000 | -.0001, .0001 |
| Total effect with Conflict as a predictor |  | **.0023*** | **.0010** | **.0003, .0043** |
| Indirect effect with Closeness as a predictor |  | -.0000 | .0000 | -.0001, .0000 |
| Total effect with Closeness as a predictor |  | .0026 | .0022 | -.0017, .0069 |
| **Independence and Self-Regulation**  **Intercept** | | | | |
| Discipline 🡪 Intercept |  | **-.0013*** | **.0005** | **-.0022, -.0003** |
| Conflict 🡪 Intercept |  | **-.0036***** | **.0005** | **-.0045, -.0027** |
| Closeness 🡪 Intercept |  | **.0133***** | **.0010** | **.0112, .0153** |
| Intercept 🡪 Self-Harm |  | **.0679**** | **.0306** | **.0078, .1279** |
| Discipline 🡪 Self-Harm |  | .0003 | .0012 | -.0020, .0026 |
| Conflict 🡪 Self-Harm |  | **.0023***** | **.0010** | **.0024, .0043** |
| Closeness 🡪 Self-Harm |  | .0026 | .0022 | -.0018, .0069 |
| Indirect effect with Discipline as a predictor |  | -.0001 | .0001 | -.0002, .0000 |
| Total effect with Discipline as a predictor |  | .0002 | .0012 | -.0021, .0025 |
| Indirect effect with Conflict as a predictor |  | **-.0002*** | **.0001** | **-.0005, -.0002** |
| Total effect with Conflict as a predictor |  | **.0020*** | **.0010** | **.0000, .0040** |
| Indirect effect with Closeness as a predictor |  | **.0009*** | **.0004** | **.0001, .0017** |
| Total effect with Closeness as a predictor |  | .0035 | .0022 | -.0008, .0078 |
| **Note.** B=unstandardized regression coefficient, SE=standard error, CI=confidence interval  p<.05*, p<.01**, p<.001*** | | | | |


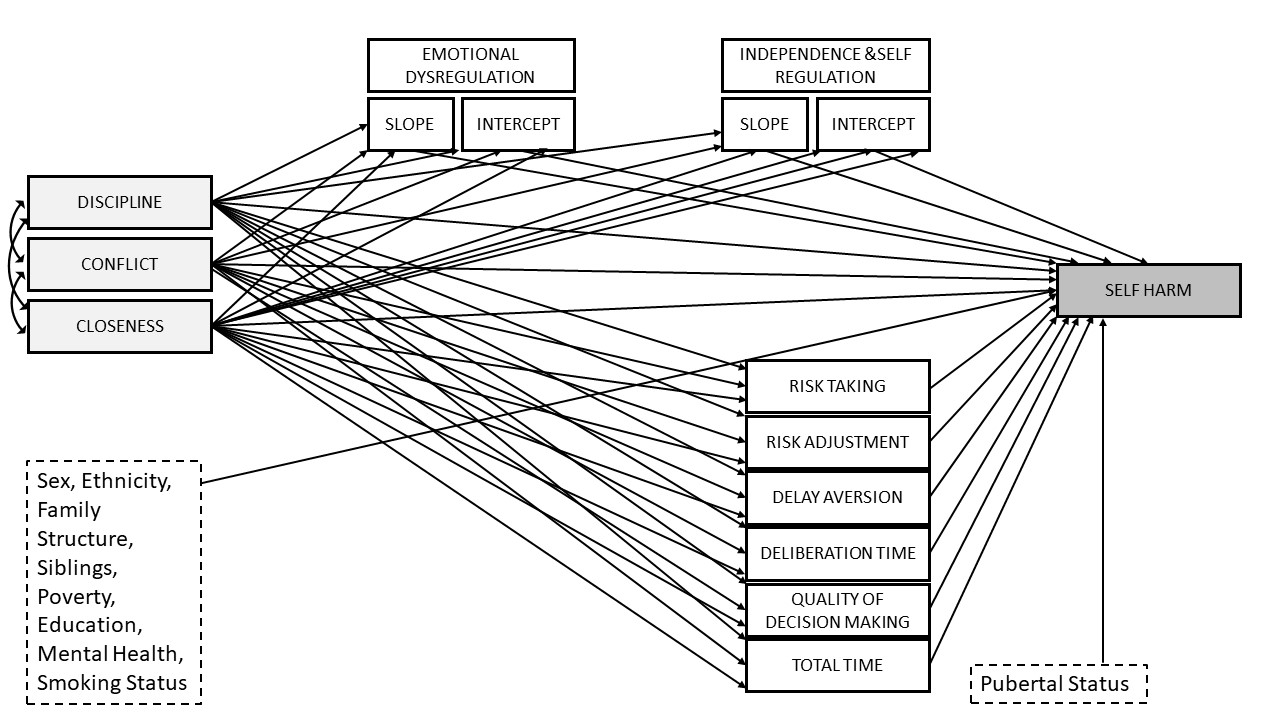


**Figure 1.** A schematic of the structural equation model of adolescent self-harm. Confounders [sex, ethnicity, family structure, siblings, parent income, parent education, parent mental health and parent smoking status] were measured at 9-months and correlated with parenting at age 3. Parenting [discipline, conflict and closeness] was measured at age 3. Emotion regulation [emotional dysregulation and independence & self-regulation] was measured at ages 3, 5, and 7, the intercept and slope within each emotion regulation variable are correlated. Decision-making and reward processing [total time, quality of decision making, deliberation time, delay aversion, risk adjustment and risk-taking] was measured using the Cambridge Gambling Task (CGT) at age 11. Pubertal status was measured as age 11 and correlated with decision-making and reward processing [CGT] at age 11. Self-harm was measured at age 14.

Table 8.

Regression coefficients for Self-Harm (adjusting for Depression at age 14) in complete and imputed cases

|  | Model 2 (n=6,463) | | | Model 2 (n=11,145) | | |
| --- | --- | --- | --- | --- | --- | --- |
|  | B | SE | 95% CI | B | SE | 95% CI |
| *Emotion Regulation* | | | |  |  |  |
| Slope of Emotion Dysregulation | **.258**** | **.092** | **.076, .439** | **.217**** | **.070** | **.079, .355** |
| Intercept of Emotion Dysregulation | .009 | .024 | -.038, .057 | .005 | .017 | -.029, .039 |
| Slope of Independence & Self-regulation | -.054 | .102 | -.255, .147 | -.061 | .077 | -.213, .091 |
| Intercept of Independent & Self-regulation | .021 | .035 | -.048, .091 | .019 | .026 | -.032, .069 |
| *CGT* | | | |  |  |  |
| Total Time | .000 | .000 | -.000, .000 | .000 | .000 | -.000, .000 |
| Deliberation Time | .000 | .000 | -.000, .000 | .000 | .000 | -.000, .000 |
| Delay Aversion | **.056*** | **.023** | **.011, .102** | **.047*** | **.019** | **.009, .083** |
| Quality of Decision Making | .054 | .042 | -.028, .138 | .046 | .029 | -.010, .011 |
| Risk Aversion | .003 | .005 | -.007, .013 | .003 | .004 | -.006, .011 |
| Risk Taking | -.045 | .031 | -.106, .017 | -.045 | .029 | -.102, .012 |
| *Parenting* | | | |  |  |  |
| Conflict | -.001 | .001 | -.003, .001 | .000 | .001 | -.002, .002 |
| Closeness | .004 | .003 | -.001, .009 | .003 | .002 | -.001, .007 |
| Discipline | **.003**** | **.001** | **.001, .005** | .001 | .001 | -.001, .002 |
| **Note.**  Model 2 = Model 1 + sex, ethnicity, birthweight, family structure, siblings, poverty, parent education, parent mental health, parent smoking status, puberty and depression  *p<.05, **p<.01, ***p<.001 | | | | | | |

We conducted additional analyses including cohort member depression (measured by the short moods and feelings questionnaire; Angold et al., 1995) at age 14 as a confounding variable. The direct effects were robust to adjustment for cohort member depression; there was a positive association between the slope of emotional dysregulation and self-harm, as well as between delay aversion and self-harm, even after adjustment for the covariates and confounders. Finally, there were significant positive associations between self-harm and sex (female), ethnicity (white), depression, parent mental health, and parent smoking.
